# Easy Rider: Monkeys Learn to Drive a Wheelchair to Navigate through a Complex Maze

**DOI:** 10.1371/journal.pone.0096275

**Published:** 2014-05-15

**Authors:** Stephanie Etienne, Martin Guthrie, Michel Goillandeau, Tho Hai Nguyen, Hugues Orignac, Christian Gross, Thomas Boraud

**Affiliations:** 1 University of Bordeaux, Institut des Maladies Neurodegeneratives UMR 5293, Bordeaux, France; 2 CNRS, Institut des Maladies Neurodegeneratives UMR 5293, Bordeaux, France; Inserm, France

## Abstract

The neurological bases of spatial navigation are mainly investigated in rodents and seldom in primates. The few studies led on spatial navigation in both human and non-human primates are performed in virtual, not in real environments. This is mostly because of methodological difficulties inherent in conducting research on freely-moving monkeys in real world environments. There is some incertitude, however, regarding the extrapolation of rodent spatial navigation strategies to primates. Here we present an entirely new platform for investigating real spatial navigation in rhesus monkeys. We showed that monkeys can learn a pathway by using different strategies. In these experiments three monkeys learned to drive the wheelchair and to follow a specified route through a real maze. After learning the route, probe tests revealed that animals successively use three distinct navigation strategies based on i) the place of the reward, ii) the direction taken to obtain reward or iii) a cue indicating reward location. The strategy used depended of the options proposed and the duration of learning. This study reveals that monkeys, like rodents and humans, switch between different spatial navigation strategies with extended practice, implying well-conserved brain learning systems across different species. This new task with freely driving monkeys provides a good support for the electrophysiological and pharmacological investigation of spatial navigation in the real world by making possible electrophysiological and pharmacological investigations.

## Introduction

Spatial learning allows people to move through their environment by acquiring, integrating and retrieving spatial information. Aspects of this include finding objects, recalling previous locations and navigating through the world [1]. To achieve this function, a cognitive map [2] needs to be created and updated in real time [3], [4]. This can be encoded either relative to an external coordinate system or to an internal frame [5], [6].

It is possible to navigate in an environment using different strategies. In rodents, the strategy used depends on the duration of training. It has been first evidenced by Packard and McGaugh, who developed a task that dissociated response (egocentric) and place (allocentric) learning strategies [7]. In this task, the rats started from the same arm of a cross maze on each trial and have to turn either left or right to another arm to obtain a reward. For a given rodent, the baited arm is always the same. The procedure is repeated several time during several days. It is possible to perform this task either by allocentric based navigation, always going to the same place in the cognitive map or egocentric based navigation, always turning in the same direction at the choice point. To dissociate the two types of learning, a probe trial in which the rat started from the opposite arm is performed. If the rat used allocentric strategy (Place Learner rat), it returned to the same arm where it had received reward. If the rat used egocentric learning (Response Learner rat), it turned in the same direction as on previous trials and ended up in the opposite to the previously rewarded arm. The ratio of place Learner to response Learner rats is higher than 1 in the early phase of the training sessions (after 7 days), while it drops to value lower than one in the late phase (after 14 days). This seminal study showed also that place strategy is dependent on the integrity of the hippocampal system while the response strategy depends on the caudate nucleus [7], [8]. A recent study in humans is in accordance with these results [9]. The authors observed that a switch from a spatial strategy to a non-spatial strategy can occur. This switch is accompanied by a drift of the intensity of the BOLD signal from the hippocampal formation to the caudate nucleus.

However, it is not clear if spatial navigation is based on the same mechanisms in primates as in rodents. The rat hippocampus contains place cells that fire in specific locations where the animal is [10]. Equivalent cells were found in non-human primates (NHP) and humans [11], but their function has been shown to be related to visual orientation which leads to some difficulties in extending the data about the dissociation between hippocampus and basal ganglia based strategies obtained in rodent to the primates.

In addition, the few studies in the NHP have been performed in virtual (VE) or restrained environments [12], [13]. This is partly due to the difficulty to allow animals to freely explore their environment because of security concerns related to the experimental protocols, but also because of the limited capacity to make electrophysiological recordings or perform pharmacological manipulations in freely moving big animals. Idiothetic cues that are integrated internally are crucial during spatial navigation [14], which limited the conclusions of VE studies.

Finally many studies compared only one non-spatial strategy, respectively, *direction* (i.e sequence of body turns) or *cue*-based (the use of local cues of the environment) strategies to the *place* strategy depending on the environment's context and distal cues. The comparison of the three alternatives (place, direction and cue) is seldom investigated [8], [15], [16].

We have therefore developed a task to study primate spatial navigation in a real world environment in which it is possible to perform pharmacological and electrophysiological procedures. This paper presents behavioral results using this platform. Three monkeys have been trained to navigate a wheelchair through a maze and have then learned to follow a route indicated by light cues. By including a probe test, we were able to discriminate between 3 distinct spatial navigation strategies, depending on the length of training.

## Materials and Methods

### Experimental subjects

Three female rhesus monkeys (Macaca mulatta) were housed under standard conditions (a 12 h light/dark cycle with light on from 7.00am to 7.00pm; humidity at 60%, temperature 22°C ±2°C). A veterinarian skilled in the healthcare and maintenance of non-human primates supervised all aspects of animal care. Experimental procedures were performed in accordance with the Council Directive of 2010 (2010/63/UE) of the European Community and the National Institute of Health Guide for the Care and Use of Laboratory Animals. The protocol received agreement from the Ethical Committee for Animal Research CE50 (Agreement number: 5012078-A). The monkeys were fed once daily and evolved in an enriched environment composed of mirrors for primates, kong toys and primatec FP3 candies (SDS Dietex, France). On experimental days water consumption was limited to the behavioral task and on non-working days monkeys received water *ad libitum*. Body weights were measured every week and maintained at a stable level during the experiments.

### Behavioral Task

#### Maze design

**Figure 1:**
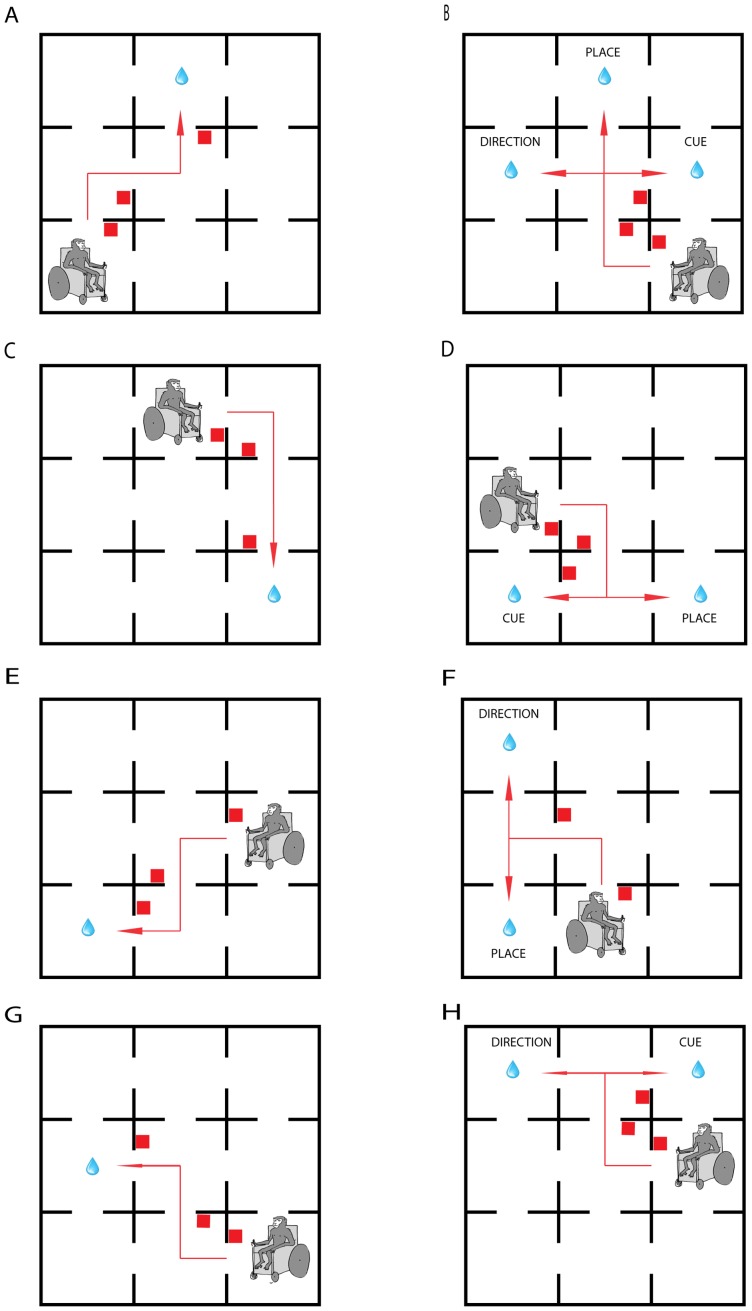
Maze and task description. The maze consists in a 3×3rooms. The rooms are not segregated by a physical door and only the room where the monkey is currently in is illuminated. The starting position for the monkey (represented by the graphic of a monkey in a wheelchair) and the reward position (represented by the drop of water) remain the same throughout a block of trials, but are modified for each block. The monkey must learn to navigate a specific path to reach the reward. After they reach the criterion (3 or 10 corrects trials in short or long training blocks respectively) animals enter a probe phase. (A; C; E; G) Acquisition phases for specific probe types, (B) Type 1 probe test (triple dissociation|, (D) Type 2 probe test (cue versus place), (F) Type 3 probe test (place versus direction), (H) Type 4 probe test (direction versus cue).

A 3×3 room maze was designed as shown in Figure 1. The maze was built into a room that could be blacked-out with heavy curtains. A smooth floor of chipboard was laid and walls constructed of 24 mm chipboard to a height of 180 cm. Each room was 150×150 cm. Walls and floors were painted matt black. There was a gap of 70 cm around the outside of the maze. Doors' width was set to 70 cm. On the left of each door opening, a set of two 7-bar LED panels (5 cm high) was placed. It was not possible to use physical doors because it would impede movement of the wheelchair. During experiments, the whole room was in darkness with blackout curtains over windows, walls and the door. The only illumination came from the LED panels. Only the LED panels in the section of the maze where the monkey was currently in were illuminated. As the room was in darkness, the monkeys could therefore not see from one section to the next. The LED panels were controlled from a central computer in a screened area of the room.

#### Pre-training

**Figure 2:**
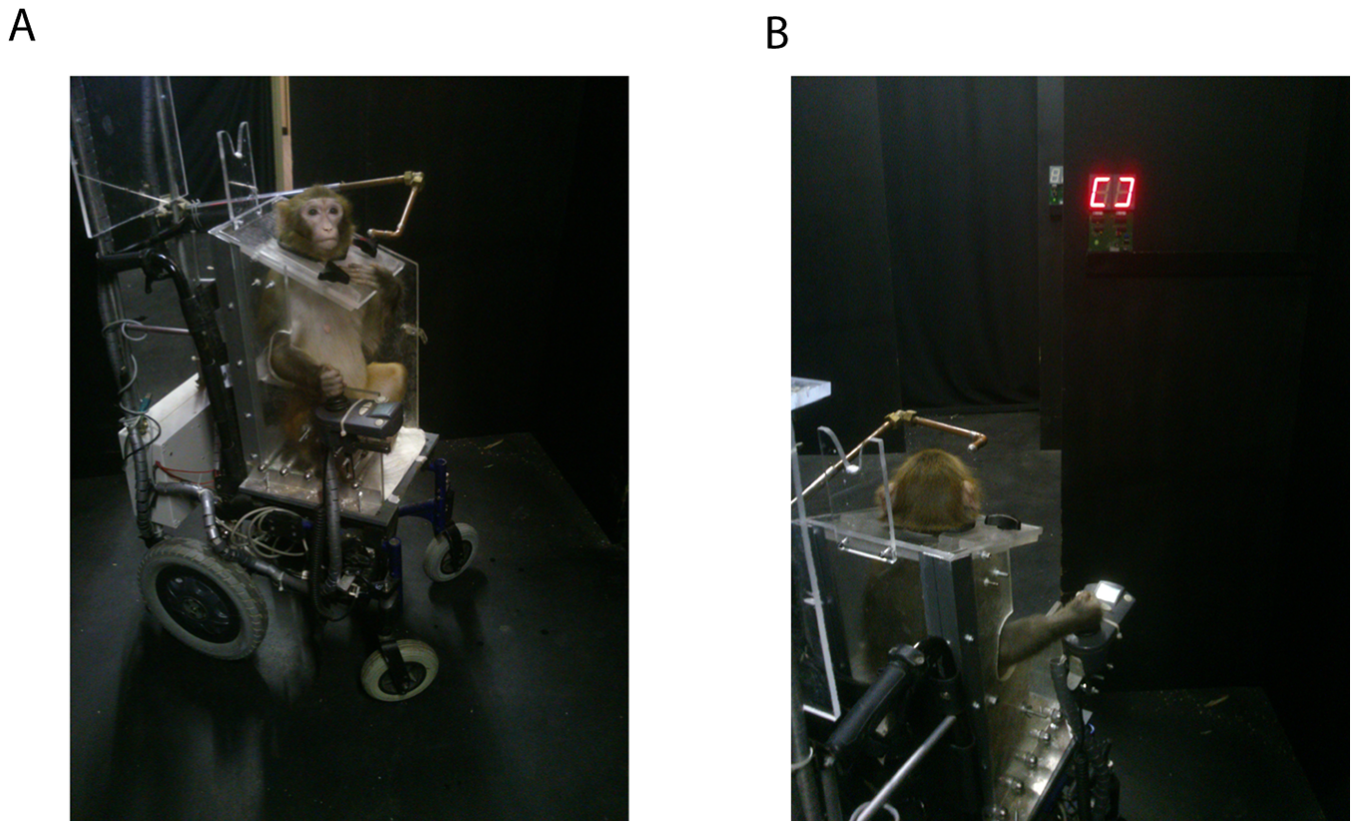
Adapted powered wheelchair. (A) The monkey sits in an electric powered wheelchair that is controlled by a joystick under the right hand, (B) the monkey learned to follow a route from room to room by choosing the one door in each room with one symbol illuminated.

In the first stage the monkeys had to be trained to drive a handicapped, young human, motorized wheelchair (Figure 2A, [Action Junior 3, Invacare]). The monkeys were first trained to use a joystick with the right hand to obtain the reward (water). They were then habituated to sitting in the wheelchair, which was controlled by a joystick on the right armrest. A restraining seat kept the monkey from leaving the chair during testing. After learning to control the chair the monkeys were then required to make the chair move in a forward direction to reach a water syringe held by an experimenter at different positions inside a room. The monkeys were then placed in the maze and taught to follow the trainer through a series of rooms to obtain a reward. They were then trained to pass through the doors in the dark maze with all symbols in the current room illuminated. Finally, they were trained to follow a route from room to room by choosing the only door in each room with illuminated panel (Figure 2B). Animals quitted the pre-training phase when they were comfortable with the wheelchair driving (no wall collision, making sequence of turns in all direction, maintaining a forward trajectory…) and finally with darkness navigation implying to make pathways by following the illuminated cue in each room.

#### Navigation task

Each session of the task consisted of two phases, an acquisition phase where the monkey has to learn a route through the maze and a probe phase where the starting position or/and the final decision point cues were changed.

During the acquisition phase, the monkeys had to learn a sequence of three choices to follow a route through a set of four rooms. At each decision point a choice was made by driving the wheelchair through a door opening into the next room. At the beginning of each trial the monkey was placed in the start room. The set of LED panels was illuminated for 2 seconds only in the start room. After this preparation period, only the symbol above the door that led to the next room in the required sequence remained lit. If the monkey chose the correct, illuminated door, when they arrived in the next room the panel in the previous room was extinguished and the set of 4 panels in the new room was lit for 2 seconds. Then, only one cue remained lit to point out the required direction. If the animal correctly followed the four rooms' sequence, a reward (water) was given in the final room. If at any point the monkey made an error (passing through any door other than the illuminated one), all panels were extinguished and the monkey returned to the start room by the handler for the next trial. After both successful and unsuccessful trials the animal was returned to the same start room by the handler in the dark by a randomly chosen route to minimize the development of the cognitive map during the inter-trial period.

The start room and the correct sequence of rooms remained the same throughout a block of acquisition trials, but were changed for each block. There were two types of acquisition block that differed only in the number of consecutive successful trials necessary to meet the criterion. In the short training block the criterion is 3 successful trials and in the long training block, it 10. At least one short block and one long block, randomly ordered, were performed each day with each monkey.

A maximum of 25 trials was allowed. If monkeys didn't meet the criterion at the 25^th^ trial, the pathway was changed or the animal was returned to his housing place depending on the experimental conditions and monkey's compliance.

When the monkey had meet the criterion a probe test was performed to determine the spatial strategy used. One of four different probe tests was used after each acquisition block, enabling the differentiation of different navigation strategies. Over twenty acquisition sessions and linked probe tests were carried out for each monkey and each acquisition criterion.

Evolution of acquisition performances (trials and errors to meet the criteria) was compared between the first 10 and the last 10 sessions.

#### Probe Tests

*Type 1: Triple Dissociation (* *Figure 1A&B* *).* During acquisition (Figure 1A) the start room and reward room have been always the same and the directions to take were signaled with the same cue on every trial. Therefore the final choice was always in the same direction and signaled with the same cue. During the probe trial (Figure 1B) both the start room and the position of the final cue were changed. The monkey followed the same cues, in the same directions, as in the acquisition phase at the first two decision points. At the final decision point (center room), there were 3 possibilities. The monkey could return to the same room in which it had received the reward in the acquisition phase (place strategy), turn left as in the acquisition phase (direction strategy) or follow the cue (cue strategy).

With only two different durations of acquisition, it was not possible to evaluate the order of all three strategies with respect to speed of learning. Therefore the following three probes were designed to test the strategies pairwise.

*Type 2: Place versus Cue (* *Figure 1C&D* *).* After the acquisition phase (Figure 1C), the start position was changed. At the final decision point (Figure 1D), the animal had to choose between returning to the same room where the reward was given during the acquisition phase (place strategy) or following the cue that had led to the reward during acquisition (cue strategy).

*Type 3: Place versus Direction (* *Figure 1E&F* *).* The animal had to choose between returning to the same room where the reward was given during the acquisition phase (place strategy) or to follow the direction that had led to reward during acquisition (direction strategy).

*Type 4: Cue versus Direction (* *Figure 1G&H* *).* The animal had to choose between repeating the same direction of turn as in the acquisition (direction strategy) or following the cue that had led to the reward during acquisition (cue strategy).

#### Data Analysis

In the acquisition phase of each block of trials, the number of errors and the number of trials the animals made to reach the criterion (3 or 10 correctly executed trials in succession) was recorded. For the probe phase, the distribution of the choices (cue, place or direction) was recorded.

Paired Wilcoxon Sign Rank, Fisher's exact and *Chi*-square's tests were used to analyze monkey's performances, distribution of choices in probe trials and the effect of the acquisition's length respectively.

Comparisons between monkeys' choices are resumed in Table 1. Data from all monkeys were pooled when no significant difference was found between them. We used a *p*-value of 0.05 as level of significance throughout this study.

**Table 1:** Monkeys' choices comparison.

Probe Test	Short Blocks	Long Blocks
Place vs Direction	0.30	0.194
Direction vs Cue	0.129	0.654
Cue vs Place	0.502	0.311

Choices distribution had been compared between monkeys (p value).

## Results

### Performances improves with learning

**Figure 3:**
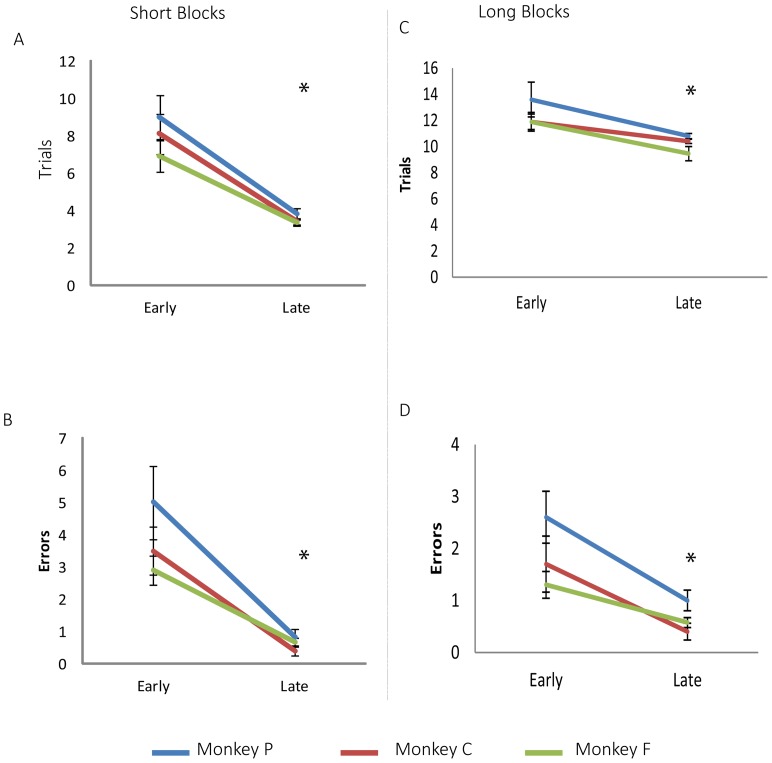
Learning rate improved with training. Early training comprised the first 10(A–B and C–D respectively) there was a significant decrease in the number of trials to reach the criterion and of the number of errors. (* p<0.05 for pooled monkeys' performances).

All the monkeys successfully learned to pilot a powered wheelchair and were able to learn to follow a path through the maze to obtain a reward. The monkey's learning performance improved with training. The number of trials to meet the criterion and the number of errors before criterion improved in both short and long acquisition blocks (Figure 3) and no difference were found between monkey's performances (Table 2).

**Table 2:** Monkeys' learning comparison.

Acquisition length	Early Training		Late Training	
	Trials	Errors	Trials	Errors
Short Blocks	0.376	0.238	0.276	0.348
Long Blocks	0.91	0.212	0.354	0.354

Monkeys performances (p values of the trials and errors to reach the two criteria) and their evolution (early versus late) were compared by using a One Way Repeated Measures ANOVA test.

### There were no significant learning strategies differences between the three monkeys

No significant difference was found between the monkeys' choices distribution in the different probe tests (Table 2).

### Strategies used can be dissociated

After criterion had been reached in an acquisition block, a probe test was conducted. Four types of probe test were used (see *Experimental Procedures*). The animal's choice at the final decision point in the probe test demonstrated which strategy the animal used to learn the acquisition pathway.

#### Type 1: Triple Dissociation

**Figure 4:**
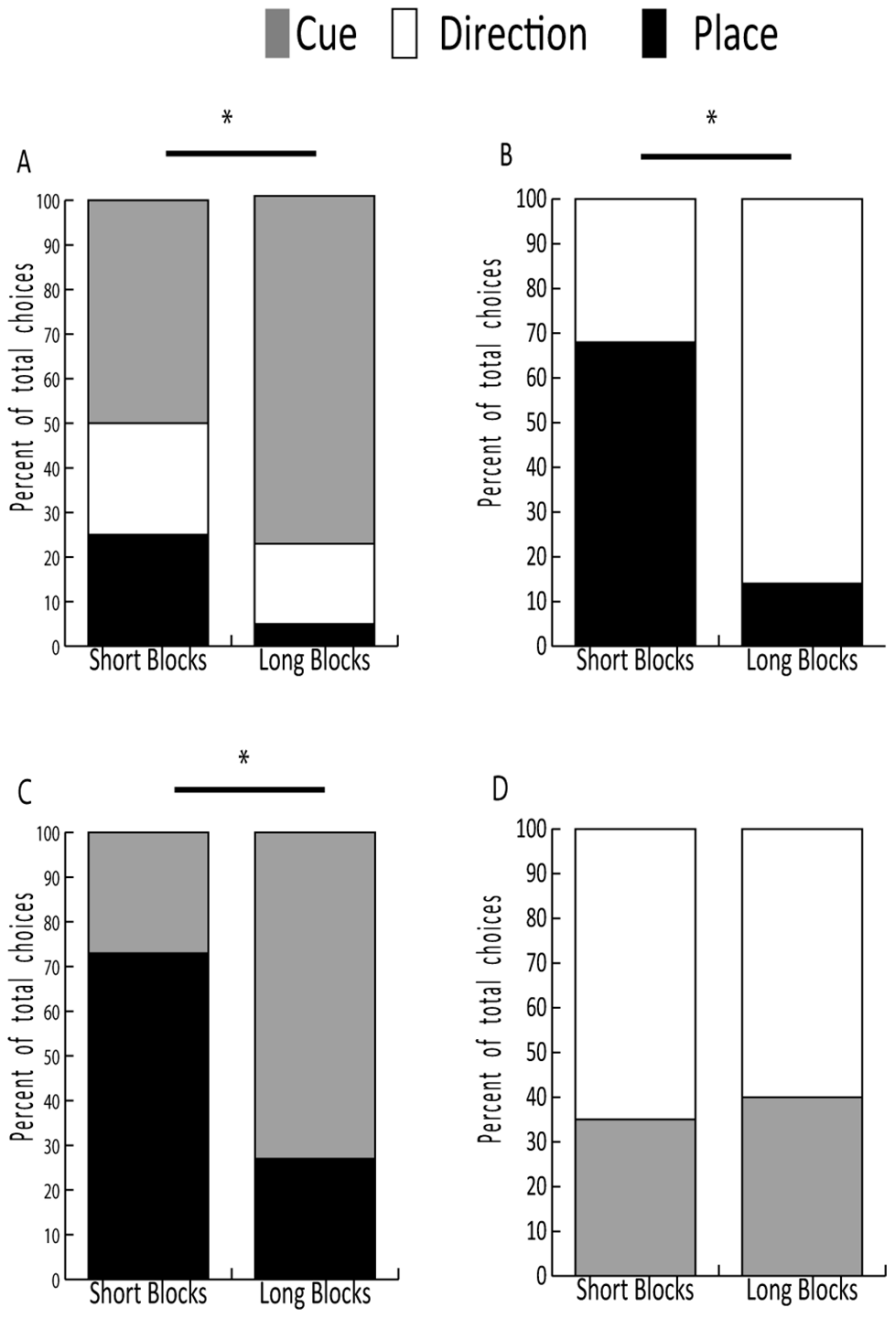
Probe Tests results: Comparisons of strategies used in probe tests in the 4 different tasks. (A) Type 1, triple dissociation, (B) Type 2, place versus direction probe; (C) Type 3, place versus cue and (D) Type 4, cue versus direction (*p<0.05).

In the type 1 probe, the animal had three choices at the final decision point (Figure 1B). After short acquisition blocks, no preference between the three possible final choices was shown. However, after long acquisition blocks, the animals made significantly more cue strategies (78%) than direction or location strategies (χ^2^ = 8.235, p = 0.016, Figure 4A).

#### Type 2: Place versus Cue

In the type 2 probe, the cue at the final decision point was moved and the animal had only two possible choices because a direction strategy would have led out of the maze, which the animals had been trained not to do (Figure 1D). There was a significant difference of navigation strategy according to the duration of the acquisition. After short blocks animals returned significantly more often to the room that was rewarded during the acquisition (place strategy), whereas after long blocks they made a cue strategy (*χ^2^* = 22.891, p<0.001, Figure 4B).

#### Type 3: Place versus Direction

In the type 3 probe, there were no cue symbols illuminated at the final choice point, so the animal could not use a cue strategy at this decision point (Figure 1F). After short acquisition blocks, animals used a place strategy more often than a direction strategy. After long acquisition blocks animals use a direction strategy more often (χ^2^ = 36.821, p<0.001, Figure 4C).

#### Type 4: Cue versus Direction

In the final probe type, it was not possible for the animal to return to the place where it had been rewarded during acquisition because this would have required a diagonal movement. When only cue and direction choices were available at the final decision point (Figure 1H), the monkeys preferentially used a direction strategy. No significant difference was found between the choices made after short and long acquisition blocks (χ^2^ = 0.117, p = 0.733, Figure 4D).

## Discussion

We present here a novel experimental test bed for the investigation of spatial navigation in primates. These behavioral results show that non-human primates are able to learn to navigate a powered wheelchair accurately through a real-world environment and that they are able to use the visual input of cues to guide the navigation. Indeed, during the acquisition phase, the monkeys were able to optimize their navigation, making virtually no errors before criterion was met.

By careful manipulation of the design of the probe phase, it was possible to distinguish between 3 different spatial navigation strategies that the monkeys used in this task: animals can return to the location where they were rewarded during the acquisition (place strategy), repeat the sequence of body turns which led to the reward room (direction strategy) or follow cue which led to the rewarded location during this acquisition (cue strategy). In the first variant of the probe (Figure 1A&B) the cue was presented in a position different from its position during the acquisition phase and the start position was changed. By forcing the final decision point to be made in the center room, there were three possible choices for the monkey, each of which implied a different strategy – place, direction or cue. The results of this triple dissociation probe type in the short acquisition blocks failed to evidence significant preference for any of the three navigation strategies. This may be an indication of the complexity of the task. The cognitive map may not be sufficiently robustly formed after only 3 consecutive correct acquisition trials to cope with both a changed starting position and a changed final decision point that gave 3 alternative choices. It is not possible to say from these results whether the animal is trying to use the cognitive map formed in the acquisition phase, but with too much competition between strategies or whether the animal has re-entered an exploration phase to form a new cognitive map. However, in the long blocks, after 10 trials, the cue navigation strategy was preferred, suggesting an increase of the predicting value of the cue and of the attention to external stimulus under uncertainty.

Based on this observation, we developed three other variants of probe test. In each of them, only two possible navigation strategies were available at the final decision point. Using these pairwise probe test variants, it was possible to demonstrate the order in which the strategies were learned. The place *vs*. cue variant probe showed a dissociation of place and cue navigation (Figure 1C&D). After short blocks animals preferentially return to the room that was rewarded during the acquisition using the place strategy, and after long blocks all animals preferentially used the cue strategy. This may indicate that the predictive value of the cue is learned more slowly than the value of a place, having a stronger influence on navigation strategy when it has been learned.

We observed the same predominance of place strategy use after short blocks in the place *vs*. direction probe (Figure 1E&F) test. However, after long acquisition blocks, animals chose the direction that they led them to the reward during the acquisition phase.

In the final probe variety, cue *vs*. direction, all animals used a direction strategy after both short and long acquisition blocks.

Based on the combination of these four probes, it seems that a place strategy is dominant early in learning and a cue/direction strategy later. In the triple dissociation probe, the cue strategy was dominant after long acquisition blocks. But when tested pairwise, no significant difference was found between the cue and direction strategies. *Direction* and *Cue* strategy can both be considered as “sequence-based” strategies, the first represented by sequence of body turns and the second relying on a distinct (or sequence of-) local landmarks [16]–[18].

These results are in line with the hypothesis that multiple, parallel neural systems are used for spatial learning [19]–[22]. These systems learn at different rates and the strategy used represents the system that arrives at a decision faster after a given length of training [21]. This is in agreement with rodent studies that have shown a transfer from goal-directed behaviors to habits implying activation of the dorsal striatum [23], [24]. A recently published review of hippocampal/basal ganglia interactions in spatial navigation, based on experimental findings in rodents, suggests three parallel loops through the basal ganglia, one of which includes the dorsal hippocampus [1]. We here present the first results showing a dissociation of three spatial navigation strategies in the non-human primate. Based on existing studies [8], [9], [25]–[29] and the theoretical 3 parallel loop architecture proposed by Retailleau et al. (2012), we propose that the cue navigation strategy observed here could be supported by a loop through sensorimotor cortex and dorsolateral striatum, the direction strategy by a loop through prelimbic cortex and dorsomedial striatum, and the place strategy by a loop through entorhinal cortex and ventral striatum that includes the dorsal hippocampus.

The test bed presented here enables the investigation of several questions that it has not been possible to consider with current experimental paradigms. Firstly, most navigation tasks in primates are performed in virtual reality environments [12], [30]. By comparing results from this task in real and virtual world environments, it may be possible to determine whether virtual world environments engage the same navigation strategies as the real world, and by extension, involve learning in the same brain areas. Despite a very challenging study point out the presence of grid cells in the primate entorhinal cortex during a virtual navigation task [31], it will be necessary to study what occurs during real environment navigation.

Two systems are known to have roles in building spatial knowledge: the hippocampal formation and the striatum. It is generally held that allocentric learning is related to the hippocampus. On the other hand, the striatum is believed to be involved in egocentric representation [32]–[35]. The role of the hippocampal formation in primates' spatial navigation is less documented than in rodents. One explanation to this lack of knowledge about primates' spatial navigation may be due to the fact it is more difficult to design a protocol which allows electrophysiological and pharmacological procedures with freely moving animals. Furthermore, BG and precisely the neostriatum involvement in spatial navigation is more and more studied [15], [16], [23], [35], [36]. Striatum can be divided into three territories (dorsolateral, dorsomedial and ventral), given this heterogeneity conclusions about each territory involvement for spatial navigation remains unclear.

Using this test bed, it should be possible to investigate the spatial learning deficits associated with animal models of neurodegenerative diseases. Electrophysiological procedures with telemetry recording system would be possible in this task. This approach will allow us to record neural activity through the 7×7 meters test room. Furthermore, inactivation of the basal ganglia in non-human primates leads to acute motor deficits. However, these animals are still able to manipulate a joystick [37], therefore it could be use to study spatial navigation deficits in animal models of both Parkinson's and Alzheimer's diseases. Finally, this task should also be readily extensible to humans for translational studies.
